# Hospital Meetings

**Published:** 1901-12-07

**Authors:** 


### HOSPITAL MEETINGS.

### HOSPITAL SUNDAY FUND.

A meeting of the Council of the Hospital Sunday Fund was held at the Mansion House on Friday last. The Lord Mayor presided, and among those present were Earl Stanhope, Sir Savile Crossley, M.P., Sir William Broadbent, Sir William Church, Archdeacon Sinclair, Prebendary Ridgeway, Sir George Bruce, Mr. R. B. Martin, M.P., Sir Edmund Hay Currie, Canon Pycke, and the Rev. Dr. Rigg. It was decided to hold the annual meeting of the clerical and lay supporters of the fund at the Council Chamber at Guildhall on Monday, December 16th, at noon, and then to recommend that the Bishop of Islington, Sir Frederick Cook, M.P., Alderman Sir Frank Green, and Mr. J. T. Scriven should be elected to fill the vacancies on the Council which had arisen during the year. On the motion of Sir Savile Crossley, M.P., it was resolved that, the attention of the Council having been called to the fact that sheet collections were sometimes made on behalf of the fund, the Council did not consider this form of collection desirable. After considering the question of the date of Hospital Sunday in next year, it was decided to recommend that Sunday, June 15th, should be selected.

### ROYAL HOSPITAL FOR INCURABLES.

The annual meeting and half-yearly election of the Royal Hospital for incurables, Putney, was held on Friday, November 29, at the Cannon Street Hotel, Mr. H. J. Allcroft, the treasurer, presiding. The report read by Mr. David W. Newton (secretary) mentioned the interesting fact that the late secretary, Mr. Frederick Andrews, who died early in the year, had bequeathed the whole of his residuary estate, amounting to about £5,000, to the institution. With regard to the funds the report pointed out that while the ordinary income was about the same as last year the legacies only amounted to £10,915, as against £18,016. Fully £32,000 was required annually to maintain the 220 inmates and 666 pensioners. The report was adopted on the motion of the Chairman, seconded by Mr. Henry Ellis. After the re-election of the retiring members of the board, Mr. Blunt proposed Miss McKee as a member of the committee of management. In the course of a long speech he said that the motion had not been brought forward with any desire to cause discord in the institution, but simply to meet what many of the subscribers considered to be a legitimate aspiration on their part, which was that at least one lady should be admitted on the board. He did not think there could be any objection as an abstract principle to adopt such a course, and, as a matter of practice, he should say that it was very necessary it should be effected in the case of a hospital where, out of 217 patients, only 36 were males. In seconding the motion, Mr. Percy Thornton, M.P., stated that although the hospital had an advisory committee of ladies, he thought that that did not go far enough, and he urged that ladies should be admitted on the board, and take their share in the management of the institution. Sir F. Dixon-Hartland, M.P., spoke strongly against the proposal, and said that the board were unanimous in the opinion that such a change would not be for the benefit of the patients or to the hospital. After further discussion the resolution, on being put to the meeting, was lost by an overwhelming majority, only 34 voting for it.

